# Food Chemicals Disrupt Human Gut Microbiota Activity And Impact Intestinal Homeostasis As Revealed By *In Vitro* Systems

**DOI:** 10.1038/s41598-018-29376-9

**Published:** 2018-07-20

**Authors:** Clémence Defois, Jérémy Ratel, Ghislain Garrait, Sylvain Denis, Olivier Le Goff, Jérémie Talvas, Pascale Mosoni, Erwan Engel, Pierre Peyret

**Affiliations:** 1 MEDIS, Université Clermont Auvergne, INRA, Clermont-Ferrand, France; 2 UR370 QuaPA, MASS Group, INRA, Saint-Genès-Champanelle, France; 3 UMR 1019, Unité de Nutrition Humaine, Equipe ECREIN, CLARA, Université Clermont Auvergne, Clermont-Ferrand, France; 4 UMR 1019, Unité de Nutrition Humaine, CRNH Auvergne, INRA, Clermont-Ferrand, France

## Abstract

Growing evidence indicates that the human gut microbiota interacts with xenobiotics, including persistent organic pollutants and foodborne chemicals. The toxicological relevance of the gut microbiota-pollutant interplay is of great concern since chemicals may disrupt gut microbiota functions, with a potential impairment of host homeostasis. Herein we report within batch fermentation systems the impact of food contaminants (polycyclic aromatic hydrocarbons, polychlorobiphenyls, brominated flame retardants, dioxins, pesticides and heterocyclic amines) on the human gut microbiota by metatranscriptome and volatolome i.e. “volatile organic compounds” analyses. Inflammatory host cell response caused by microbial metabolites following the pollutants-gut microbiota interaction, was evaluated on intestinal epithelial TC7 cells. Changes in the volatolome pattern analyzed *via* solid-phase microextraction coupled to gas chromatography-mass spectrometry mainly resulted in an imbalance in sulfur, phenolic and ester compounds. An increase in microbial gene expression related to lipid metabolism processes as well as the plasma membrane, periplasmic space, protein kinase activity and receptor activity was observed following dioxin, brominated flame retardant and heterocyclic amine exposure. Conversely, all food contaminants tested induced a decreased in microbial transcript levels related to ribosome, translation and nucleic acid binding. Finally, we demonstrated that gut microbiota metabolites resulting from pollutant disturbances may promote the establishment of a pro-inflammatory state in the gut, as stated with the release of cytokine IL-8 by intestinal epithelial cells.

## Introduction

People are exposed on a daily basis to a variety of environmental pollutants arising from industries, transports, heating or agriculture. Persistent organic pollutants (POPs), such as polycyclic aromatic hydrocarbons (PAHs), polychlorobiphenyls (PCBs), brominated flame retardants (BFRs), polychlorinated dibenzo-p-dioxins (PCDDs) and pesticides, are compounds that cause concern because of their toxicity, persistence in the environment, capacity to move over very long distances and ability to accumulate in organisms^1^. Exposure to these pollutants has been linked to various pathologies, including metabolic^2^, immune^3,4^ and reproductive disturbances^5^ and even cancers^6,7^. Heterocyclic amines (HCAs) are foodborne chemicals produced by some cooking practices that are similar to POPs in terms of structure and toxic properties. HCAs are mutagenic and characterized as possible human carcinogens^8^, increasing the risk of the emergence of colorectal cancer^9^.

Because exposure to POPs and foodborne chemicals occurs mainly through the diet, the host gastrointestinal tract (GIT) and the gut microbiota are likely to be exposed to these compounds. Recent studies have shown that xenobiotic-microbiota interactions may lead to modifications of the gut microbiota composition and functions, which could then impact host homeostasis^10,11^. In a murine model, Zhang and colleagues have shown that five days of orally administered 2,3,7,8-tetrachlorodibenzofuran (TCDF) lead to dramatic modifications of the structure of the mice gut microbiota by reducing the ratio of Firmicutes to Bacteroidetes, accompanied by activation of the microbial fermentation (elevation of short chain fatty acids in feces and cecal content extracts)^12^. Conversely, chronic exposure (26 weeks) of the mouse gut microbiota to 2,3,7,8-tetrachlorodibenzo-p-dioxin (TCDD) induced a major increase in the Firmicutes to Bacteroidetes ratio, while the same exposure on a short time scale (2 days) did not appear to have any significant effects^13^. To complement these observations, a recent study showed an increase of 13 antimicrobial resistance genes and 1 mobile genetic element gene along with a bloom of *Enterobacteriaceae (bacterial groups harboring these genes)* within 8 days of TCDD exposure in the murine gut microbiome^14^. Finally, acute exposure to a mixture of PCBs or benzo[*a*]pyrene (B[*a*]P) induced either a substantial decrease in the level of Proteobacteria in the mouse gut microbiota or showed no significant impact on the human gut microbiota but induced a shift in microbial metabolic activity, respectively^15,16^.

The gut microbiota may, in return, metabolize chemical compounds, which might deflect their therapeutic (drugs) or toxic (environmental and foodborne chemicals) properties toward the host^17,18^. A previous work reported that six commonly used host-targeted drugs, induced 328 microbial genes, most of which could be associated with drug transport or degradation^19^. Dichlorodiphenyltrichloroethane (DDT), an organochlorine insecticide, has been found to be metabolized to dichlorodiphenyldichlorophenylethane (DDD) by rat and human fecal microbiota, although it remains unclear whether this biotransformation corresponds to bioactivation or detoxification because both DDT and DDD are probable endocrine disruptors in humans^20^. Finally, the human gut microbiota has been shown to biotransform B[*a*]P and 2-amino-1-methyl-6-phenylimidazo[4,5-b]pyridine (PhIP) into 7-hydroxybenzo[*a*]pyrene and 7-hydroxy-5-methyl-3-phenyl-6,7,8,9-tetrahydropyrido[3′,2′:4,5]imidazo[1,2-a]pyrimidin-5-ium chloride (PhIP-M1) identified as B[*a*]P and PhIP derivatives, respectively^21–23^.

Beside metabolomics where all metabolites produced by living organisms are analyzed, volatolomics focuses on the study of volatile metabolites reducing the complexity of the analysis. This method has proven to be a promising omic approach to diagnose metabolism changes in response to physiological stresses induced by pathology^24^ or xenobiotic exposure^25^. Regarding gastrointestinal or inflammatory disorders, Ahmed *et al*. reported that changes in fecal VOC pattern may result from changes in the microbiota and/or pathologies in the GIT^26,27^. Recently, a previous work from Defois *et al*. showed *in vitro* that volatolomics along with metatranscriptomics enable to decipher a rapid change in the gut microbiota activity following acute exposure to B[*a*]P while the gut microbiota composition remained stable^16^.

In the present work, we characterized within *in vitro* systems the impact of six POPs and/or foodborne chemicals that are frequently found in the diet and considered them as models for the effects of various classes of pollutants on human gut microbiota functions at the metatranscriptome and volatolome levels. We also measured the inflammatory properties of the metabolites arising from this xenobiotic-gut microbiota interaction to characterize their effects on gut homeostasis.

## Results

### Pollutant exposure alters the fecal microbiota volatolome pattern

Analysis of the microbial volatolome is a promising approach to detect an imbalance of microbial activity. Five pollutants (TCDD, deltamethrin, HBCD, B[*a*]P and PhIP) and one mixture of pollutants (PAHs) were screened for their impact on human fecal microbiota functions following a 24-hr exposure. Briefly, the pollutants were added at different concentrations into Hungate tubes along with a fecal microbiota suspension containing human *in vitro*-cultured feces. Volatolome patterns were assessed by solid-phase microextraction coupled to gas chromatography-mass spectrometry (SPME-GC-MS) analysis and vehicle 1 and 2 conditions (methanol and methanol:dichloromethane mixture, respectively) were conducted to remove the effects of the vehicles on the microbial community.

More than 250 volatile organic compounds (VOCs) were detected by SPME-GC-MS following the 24 hr of pollutant exposure. We identified 5, 2, 7 and 4 VOCs that were significantly altered by deltamethrin, PhIP, TCDD and PAHs compared to their respective vehicle condition (Table 1). Sulfur compounds (including thioesters) were increased in samples exposed to deltamethrin and TCDD conditions with 3 and 5 compounds, respectively. One ketone (2,2,4,4-tetramethyl-3-pentanone) decreased in deltamethrin, PhIP and TCDD conditions and one hydrocarbon (m- or p-xylene) decreased in deltamethrin and PhIP conditions. Following PAHs exposure, one phenol (4-methylphenol), one ester (propylphenylacetate) and one ketone (methylacetophenone) increased while one unknown compound decreased. Finally no significantly altered compounds were found following B[*a*]P and HBCD exposure.

**Table 1:** Volatile metabolites detected in the fecal microbiota volatolome as significantly altered by the pollutants. DeltaM: deltamethrin.

Volatile Metabolite	m/z^a^	LRI^b^	ID^c^	*p*-value^d^				Ratio (Pollutant / Vehicle)			
				DeltaM	PhIP	TCDD	PAHs	DeltaM	PhIP	TCDD	PAHs
**Sulfur compounds**											
Carbon disulfide	76	<600	a,b			7.4E-04				3.80	
Dimethyl disulfide	94	748	a,b	2.3E-03		1.8E-03		1.69		2.14	
Dimethyl trisulfide	126	983	a,b	2.5E-03		5.5E-05		1.78		2.39	
4- or 5-methyl-2-acetylthiazole	126	1116	a,b			2.3E-03				1.51	
Dimethyl tetrasulfide	79	1243	a,b	1.0E-03		9.6E-04		3.68		6.21	
**Thioesters**											
S-methyl 3-methylbutanethioate	85	945	a			2.3E-03				1.46	
**Phenols**											
4-methylphenol	105	1076	a,b				3.0E-04				1.30
**Esters**											
Propylphenylacetate	91	1345	a,b				3.0E-03				2.86
**Ketones**											
2,2,4,4-tetramethyl-3-pentanone	85	939	a,b	4.0E-06	0.0E + 00	2.0E-06		0.52	0.56	0.53	
Methylacetophenone	119	1198	a,b				2.2E-04				1.61
**Hydrocarbons**											
m- or p-xylene	91	875	a,b	4.0E-04	2.1E-04			0.38	0.38		
**Unknown**											
Unknown	57	<600					8.3E-04				0.37
Total^e^								5	2	7	4

^a^Mass fragment used for peak area determination.

^b^Linear retention index on a RTX-5MS capillary column.

^c^Tentative identification based on (a) mass spectrum, (b) linear retention index from the literature.

^d^*P*-values corrected for multiple testing.

^e^Total number of volatiles significantly altered by the pollutants.

### Pollutant exposure alters the fecal microbiota metatranscriptome

The volatolome analysis showed that four tested chemicals (deltamethrin, PhIP, TCDD and PAHs) significantly shifted the microbial volatile pattern, suggesting that the activity of the microbiota might have been affected. Thus, using RNA-sequencing, we investigated the microbial response to chemicals by analyzing the differential metatranscriptome between exposed and non-exposed microbiota and then deducing the metabolic pathways that may have been altered.

**Figure 1:**
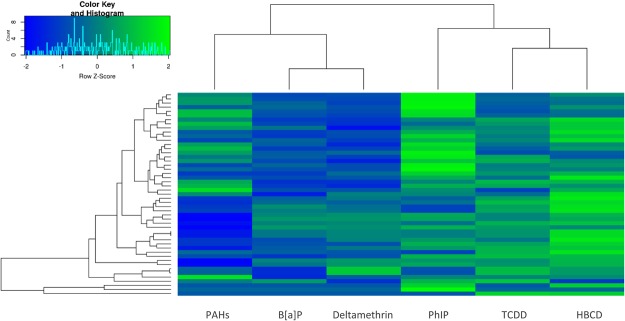
Transcript levels related to microbial GO slim terms that were up and downregulated after the 24 hr of pollutant exposure. Analysis was performed on the pooled rRNA-depleted RNA arising from five technical replicates. Variations are expressed as the Z-Score. Lines represent GO slim terms, columns represent pollutant samples.

**Figure 2:**
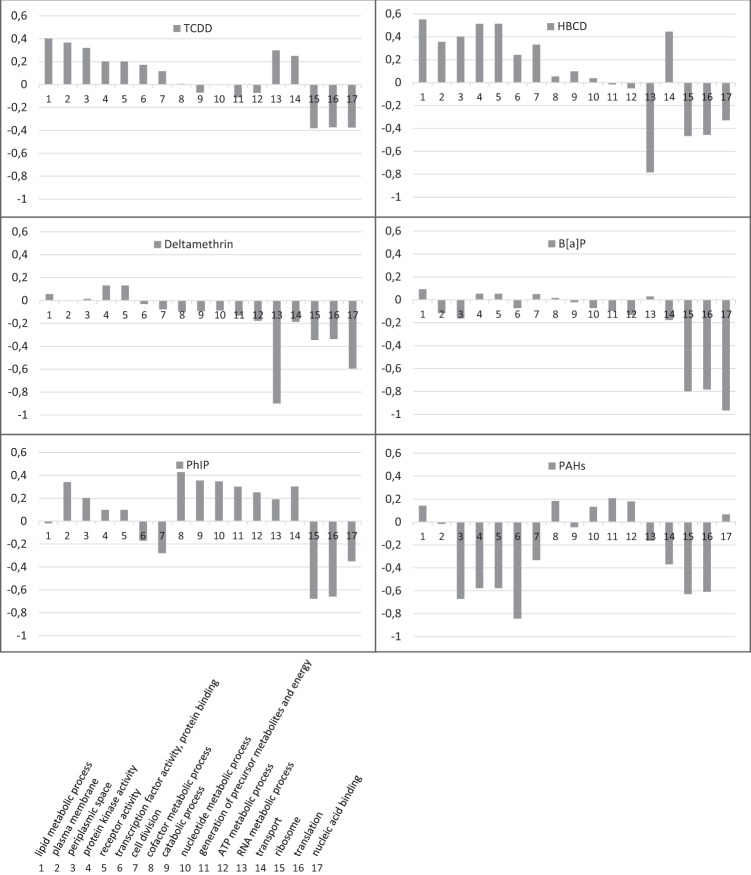
Transcript levels related to microbial GO slim terms that were up and downregulated after the 24 hr of pollutant exposure. Variations are expressed as the log2 of the pollutant and the vehicle CPM abundance ratio (y-axis). Analysis was performed on the pooled rRNA-depleted RNA arising from five technical replicates. GO slim terms are represented on the x-axis. GO: gene ontology.

Functional assignation was realized at the gene ontology (GO) slim term and at the gene family levels, and the results are presented as copies per million units (CPM) abundances. To discard the effects of both vehicles on the fecal microbiota, the pollutant samples were directly compared to the corresponding vehicle samples. Following the 24 hr of exposure, variations observed at the GO level clustered the pollutant samples into two main groups (Fig. 1). The PAH, B[*a*]P and deltamethrin samples showed a strong downregulation of GO slim terms together with a weak upregulation of slim terms, whereas the PhIP, TCDD and HBCD samples showed a strong upregulation of GO slim terms together with a weak downregulation of slim terms. Although each pollutant induced specific transcriptomic responses, general trends were largely shared by the six pollutant samples, such as an increase in transcript levels related to the lipid metabolism process, plasma membrane, periplasmic space, protein kinase activity and receptor activity. Conversely, a decrease in transcript levels related to ribosome, translation and nucleic acid binding was observed (Fig. 2).

**Figure 3:**
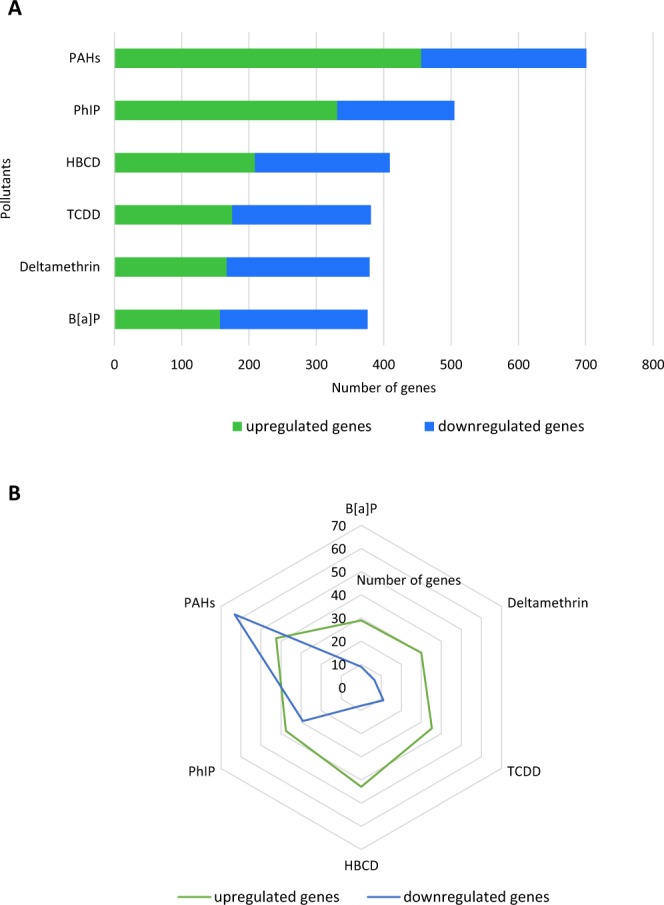
Differentially expressed microbial genes after the 24 hr of pollutant exposure. (**A**) Number of differentially expressed genes. (**B**) Mean abundances in CPM of the differentially expressed genes. Analysis was performed on the pooled rRNA-depleted RNA arising from five technical replicates. Only genes with at least a 3-fold change are represented, and values were derived from a comparison between the pollutant and the vehicle condition.

At the gene family level, the number and mean abundance of the differentially expressed genes (>3-fold change) differed among the pollutant samples (Fig. 3). The number of upregulated genes varies from 157 to 456 for the B[*a*]P and PAH exposure, respectively. The number of downregulated genes varied from 174 to 245 for the PhIP and PAH exposure, respectively (Fig. 3A). While the mean abundance of upregulated genes was within a similar range for all samples (from 29 CPM for the B[*a*]P to 43 for the HBCD samples), the mean abundance of downregulated genes was far higher for the PAH sample (from 7 CPM for the deltamethrin to 63 for the PAH samples) (Fig. 3B). The high mean abundance of downregulated genes in the PAH sample might explain the substantial downregulation observed for some GO slim terms (Fig. 1).

Among the differentially expressed genes, we identified three rubredoxin (Rdx)-coding genes that were found in the most upregulated genes in the PhIP and PAH samples compared with their respective vehicle samples (27 to 437-fold change) (Supplementary Data 1). The products of this gene family have been implicated in the alkane degradation pathway, which is part of hydrocarbon metabolism^28,29^.

**Figure 4:**
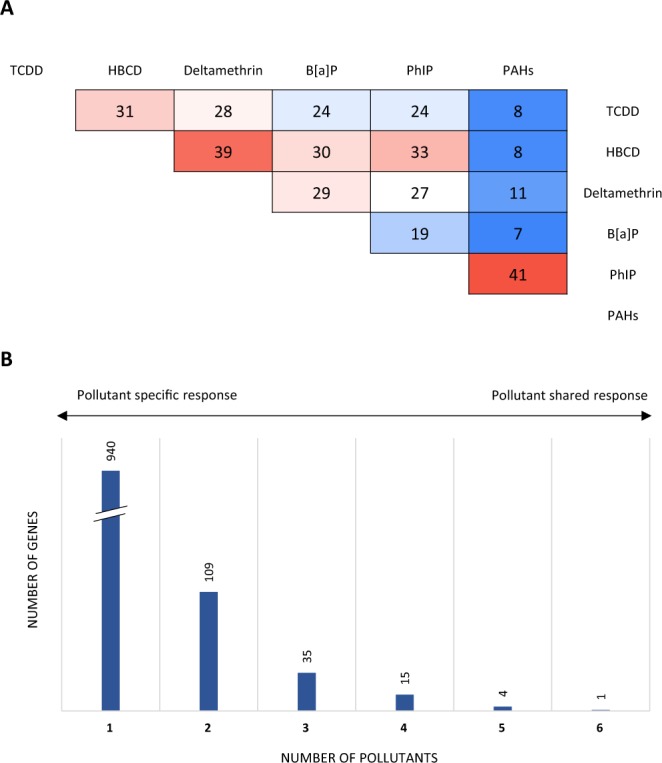
Microbial genes specifically induced by pollutants after the 24 hr of exposure. (**A**) Number of pollutant-specific genes shared between each couple of pollutants. (**B**) Representation of the core pollutant-specific genes. Analysis was performed on the pooled rRNA-depleted RNA arising from five technical replicates.

Some genes were found to be specifically induced by the pollutants (not expressed in their respective vehicle condition) and will be referred as “pollutant-specific genes” (Supplementary Data 2). PhIP and PAHs shared the largest number of pollutant-specific genes (41 genes), highlighting common microbial metabolic pathways activated by the exposure (Fig. 4A). Conversely, the PAH sample shared few pollutant-specific genes (7 to 11 genes) with the other chemicals (except with PhIP). We identified 109 to 1 pollutant-specific genes shared by 2 to 6 of the chemicals, respectively (Fig. 4B and Supplementary Data 2). Among the pollutant-specific genes, a Rdx-coding gene (different from the three others previously described) was the most highly induced gene by the HBCD (344 CPM). Finally, a gene that was specifically induced by the PhIP and PAH pollutants encodes an uncharacterized protein harboring the Toluene_X Outer Membrane Transport family domain (UniRef50_R5K1C5). Proteins of this family are involved in toluene catabolism and the degradation of aromatic hydrocarbons. One gene that was specifically expressed in response to the 6 pollutants was found to be an uncharacterized protein-coding gene (UniRef50_R7EHV5) (Supplementary Data 2).

### Fermentation-derived supernatants do not induce TC7 cell death

As shown above, pollutant exposure modified the microbial activity and thus modified the compounds produced in the fermentation-derived supernatants (FDS). We can assume that the microbial community may also metabolize the pollutants and convert them into more or less harmful compounds towards the host. These compounds may induce cell damage in the intestinal epithelium, potentially through the release of inflammatory molecules.

**Figure 5:**
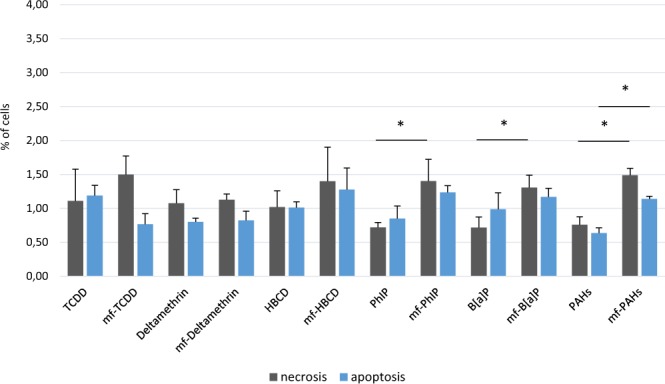
Percentage of necrotic and apoptotic TC7 cells after 4 hr of FDS and microbiota-free medium exposure. Values are the mean of the three replicates ± SEM. Significant variations were assessed using the Mann-Whitney test (p-value < 0.05). mf: microbiota-free.

To test these hypotheses, we exposed TC7 cells to FDS for 4 hr. Cells were analyzed by flow cytometry to assess their viability, and the cell culture supernatant was harvested to quantify pro (IL-8, TNFα) and anti (IL-10) inflammatory cytokine release. No differences in the proportion of necrotic or apoptotic cells were observed between the pollutant and vehicle samples. Only exposure to 10% DMSO, a necrotic and apoptotic reagent used as positive control, led to a significant increase in both necrotic and apoptotic cells, making up 25.7% and 7.9% of the total cells, respectively (Supplementary Figure 1). FDS was also compared to fecal microbiota-free colon medium supplemented with each pollutant at its initial experimental concentration. Without the fecal microbiota, a slight increase in necrotic (and apoptotic for PAHs) cells was observed for the PhIP, B[*a*]P and PAH samples (Fig. 5). Necrotic cells increased from 0.72, 0.72 and 0.76% to 1.40, 1.31 and 1.49% in the PhIP, B[*a*]P and PAH samples, respectively. Apoptotic cells in the PAH sample increased from 0.64 to 1.14%.

### Fermentation-derived supernatants alter IL-8 production in TC7 cells

IL-8, TNFα and IL-10 release was measured in TC7 cell supernatants to highlight a putative pro- or anti-inflammatory cell response.

**Figure 6:**
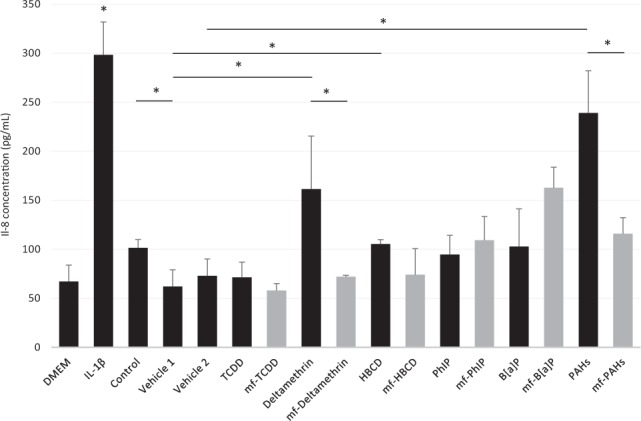
IL-8 release in the TC7 cell culture supernatants. TC7 cells were exposed to FDS and microbiota-free medium for 4 hr. Values represent the mean of three replicates ± SEM. Significant variations were assessed using the Mann-Whitney test (p-value < 0.05). DMEM was a negative control for toxicity and inflammation, whereas IL-1β was a positive control for inflammation in TC7 cells. Control: no pollutant and no vehicle; Vehicle 1: methanol; Vehicle 2: methanol:dichloromethane 1:1; mf: microbiota-free.

The vehicle samples did not induce the release of IL-8 compared with DMEM (Fig. 6). However, the control condition induced a slight significant increase in IL-8 compared with the vehicle 1 condition (62.1 to 101.5 ng/mL). The deltamethrin, HBCD and PAH samples showed induction of a significant increase in IL-8 compared with their associated vehicle sample. The strongest increase was observed for the PAH sample, which exhibited a 3.85-fold increase (from 62.1 to 239 ng/mL). The responses to PAHs and the IL-1β (pro-inflammatory control) were similar, with 239 and 298 ng/mL of IL-8 release. Compared with the microbiota-free conditions, significant variations were observed for the deltamethrin and PAH samples, with, on average, a 2-fold increase. Interestingly, the PhIP and B[*a*]P samples showed induction of less IL-8 release compared with the microbiota-free condition, but these variations were not significant. In contrast to IL-8, TNFα and IL-10 were not detected in any of the culture cell supernatants.

## Discussion

Food pollution by chemicals is of great concern due to the toxicity of the compounds that accumulate in the food chain and because the exposure occurs chronically throughout life. While the toxicity toward the host has been evaluated for some representatives of the main groups of contaminants, their impact on the gut microbiota has received less attention. In this work, we investigated the impact of six pollutants on human gut microbiota activity at the volatolome and metatranscriptome levels. The microbial- and chemical-derived compounds generated during acute (24 hr) exposure of the microbiota to the pollutants were then assessed in TC7 cell monolayers to characterize their toxicity and inflammatory properties in the host.

The doses used in this work are lower or in the range of previous studies carried out to examine the impact of TCDD, PhIP and B[*a*]P on the gut microbiota, allowing us to make comparisons and generate discussions^13,14,16,21,22^. The doses used for PAH, HBCD and deltamethrin exposures were far lower than those used for toxicity studies in mice^30–32^. The doses used in this study are still higher than the expected daily consumption. However, the purpose of this study was to investigate a unique exposure to pollutants that might provide initial insights into their effects on the human gut microbiota. Of course, further studies will be necessary to mimic the chronic exposure that occurs throughout a person’s life.

The study of the physiology of the GIT and the role of the gut microbiota is difficult in humans due to ethical (e.g. exposure to pathogens, chemicals, pharmaceuticals) and technical (difficulty to access the GIT) aspects, most often limiting studies to fecal analysis (non-invasive, cheap). Our work was based on *in vitro* systems thus allowing us to decipher the direct impact of the pollutants on the gut microbiota. Such systems have been used in various applications such as the fermentation of food components^33–35^, the impact of antibiotics on the microbiota of patients with Crohn’s disease^36^, the metabolism of gut methanogenic archaea^37^ or the degradation of toxic compounds such as PAHs by the gut microbial metabolism^21^. Meanwhile these models are missing the host counterpart, thus the results arising from this work should be considered carefully until *in vivo* experiments are conducted.

VOCs are small molecules produced by living organisms that play an important role in chemical ecology, specifically in the biological interactions between organisms^38^. During the past decade, VOCs have gained recognition in the health care field because they are presently used as biomarkers to detect various human diseases, including cancers^39,40^. In this work, we analyzed the volatolome pattern of the fecal microbiota after 24 hr of pollutant exposure. Overall, a partially shared response between deltamethrin and TCDD exposure was observed, with an enrichment of the microbial volatolome with sulfur compounds and a decrease in one ketone compound (2,2,4,4-tetramethyl-3-pentanone) while following PhIP and PAHs exposure the volatolome patterns appeared more specific (Table 1). Surprisingly we observed no significant shift in the VOC pattern following HBCD and B[*a*]P exposure contrary to a previous work in which two human fecal microbiota were exposed to B[*a*]P at three gradual concentrations^16^. More precisely, in the first study, at the same concentration that was used in the present work, a decrease in 1 ketone and 1 ester compound was observed. However, while not significant (*p*-value< 0.05 but >0.05/ni), similar variations have been highlighted in the present work following B[*a*]P exposure including the decrease of 1 ketone and 9 ester compounds (data not shown). Until now, studies examining microbial VOCs have been scarce, and only a few available databases have described VOCs, their emitting organisms and their biological activities^41,42^. Despite limited information on VOC biological activities, some links between microbial VOC patterns and many physiological and pathological states have been reported, including gastrointestinal metabolic disorders^43,44^. A decrease in volatolome ketones has recently been reported in patients with an inflamed gut (Crohn’s disease, ulcerative colitis or pouchitis)^44^. Moreover, patients with Crohn’s disease showed increased production of phenol, as observed herein following PAHs exposure. However, sulfur compounds, which increased in this study, decreased in patients with Crohn’s disease. As alterations in the microbial VOC patterns are a consequence of the disruption of the normal bacterial ecology in pathologies such as inflammatory bowel disease (IBD), it can be hypothesized that similar disturbances (shift in VOC profiles) are triggered by chemical agents, such as environmental pollutants.

To obtain a more detailed characterization of the altered metabolic pathways, we analyzed gut microbiota gene expression using RNA sequencing. The variations observed at the GO level were pollutant-dependent; however, general trends emerge from the analysis, showing an increase in transcript levels related to the lipid metabolism process, plasma membrane and periplasmic space following dioxin, brominated flame retardant and heterocyclic amine exposure. A previous study from the laboratory also identified an increase in these metabolic pathways after a 24-hr B[*a*]P exposure^16^, indicating a clear impact of such hydrophobic compounds on cell membranes. It has been proposed that lipophilic compounds, when crossing membranes, increase the membrane fluidity, leading to a loss of membrane functionality^45^. One of the major adaptive mechanisms of bacteria cells to counteract this effect is to increase their membrane rigidity (enhance membrane lipid saturation) to prevent compound accumulation within the cell^46^.

We also identified an increase in transcript levels related to protein kinase activity and receptor activity mainly for TCDD, HBCD and deltamethrin exposure. Such variations have never been identified elsewhere following environmental toxicant exposures. However, the increase in the transcript levels of these two GO slim terms might indicate an increase in the cell signaling network and thus an activation or repression of cellular responses due to the presence of the pollutants.

A decrease in transcript levels related to the GO slim terms ribosome, translation and nucleic acid binding was observed. In a recent study, we identified a reduction in energy metabolism following B[*a*]P exposure and proposed that the bacterial cells might engage adaptive processes to manage stressful events. Microorganisms may thus engage energy for adaptation mechanisms to ensure necessary physiological functions prior to energy expenditure for growth^46^. Ribosome assembly and protein synthesis processes are major targets for a large number of antibiotics^47^. This observation potentially raises the hypothesis that POPs may act as antibiotics targeting the bacterial translation apparatus. Interestingly, a > 2-fold increase in 13 antimicrobial resistance genes was observed in the murine gut microbiome within 8 days of TCDD exposure^14^. Among these genes, some (multidrug-resistant genes) were associated with resistance to antibiotics targeting bacterial protein synthesis (aminoglycoside, glycylcycline, macrolide and erythromycin).

Rubredoxin-coding genes have been found to be highly induced in PhIP and PAH samples and found specifically (absence of the vehicle condition) in the HBCD sample. Rdxs are essential electron transfer components of bacterial membrane-bound alkane hydroxylases found in aerobic n-alkane degradation pathways^28,48^. However, in anaerobic organisms, Rdxs have been identified as crucial for oxidative stress responses (reduction of oxygen or reactive oxygen species)^49^.

Interestingly, a gene encoding an unknown protein carrying a Toluene_X Outer Membrane Transport family domain was identified as specific to the PhIP and PAH samples. This protein domain is found in membrane proteins with an uncharacterized function that are involved in toluene catabolism and the degradation of aromatic hydrocarbons^50,51^. Hence, our result highlights the potential metabolism of PhIP and PAH compounds by the human gut microbiota. This result is in agreement with previous studies showing that the gut microbiota is capable of metabolizing these chemical compounds into 1-hydroxypyrene, 7-hydroxybenzo[*a*]pyrene and PhIP-M1 as pyrene, benzo[*a*]pyrene and PhIP metabolites, respectively^21–23^.

Gut microbial fermentation products play a major role in human health and disease^52^. An imbalance of these compounds (mediated by altered microbial activity) may directly or indirectly impact the gut environment and the host. However, although the microbial metabolism of xenobiotics, such as POPs, has been demonstrated, the toxicity of their metabolites remains unknown. Thus, we investigated the impact of FDS on the TC7 cell line by measuring the induction of the necrotic and apoptotic processes, along with the release of inflammatory cytokines after 4 hr of contact.

The FDS did not specifically induce necrosis or apoptosis in the TC7 cell line under these experimental conditions. Moreover, the microbiota seemed to limit the necrotic effect of PhIP, B[*a*]P and PAHs. Thus, the gut microbiota could play a protective role for the host against some toxic compounds. At this stage, it is not possible to precisely identify the protective mechanism and discriminate between a potential pollutant degradation or sequestration by the microbiota. No necrotic or apoptotic effects have been observed for the other pollutants under our experimental conditions. We cannot exclude toxic effects after prolonged contact periods and/or with other cell lines. Concerning the inflammatory response, FDS from deltamethrin, HBCD and PAH samples induced a significant increase in IL-8 production by TC7 cells. Our assumption is that pollutants induce a functional dysbiosis that modifies the balance between anti-inflammatory and pro-inflammatory metabolites in the supernatant. By contrast, we also observed that the microbiota that were in contact with pollutants, such as PhIP and B[*a*]P, could slightly reduce the release of IL-8. In this case, the microbiota could also play a protective role against inflammatory induction by chemical compounds, but as mentioned previously, we could not determine the involved mechanisms (pollutant degradation and/or sequestration, production of anti-inflammatory metabolites). However, it must be considered that previous *in vivo* studies showed the establishment of a pro-inflammatory intestinal environment following chronic B[*a*]P exposure at high doses^53,54^. B[*a*]P has also been shown to potentiate murine intestinal inflammation caused by a high-fat diet^55^.

TNFα and IL-10 cytokines were not detected in any of the culture cell supernatants. These two cytokines have been previously detected in Caco-2 experiments; however, immune cells^56,57^ and/or bacterial cells^58,59^ seem to be obligate partners to induce their expression and release. Other experimental conditions, such as cellular differentiation and incubation time, must be taken into account because the cytokine expression varies depending on these parameters. As an example, the general inflammatory response is higher in proliferating cells compared with fully differentiated cells^60,61^.

Chronic pollutant expositions may progressively induce a low-grade inflammatory status. To our knowledge, no data are available regarding the inflammatory properties induced by POPs in intestinal epithelial cells. However, environmental chemicals have been characterized as triggering a pro-inflammatory response mediated by the aryl hydrocarbon receptor (AhR) in different cell types^62–64^. The AhR is a cytosolic transcription factor that is activated by numerous environmental hydrophobic chemicals, leading to their metabolic clearance and detoxification. While the AhR is often regarded as a xenobiotic receptor, it is becoming increasingly clear that this receptor exhibits activity that influences numerous endogenous functions, including immune functions^65,66^. For example, Kobayashi and colleagues have shown that environmental exposure to TCDD, through binding to AhR, exacerbates rheumatoid arthritis pathophysiology *via* stimulation of the NF-κB and ERK signaling pathways^67^. The rodent AhR homolog is known to bind TCDD and PAHs with ~10-fold higher affinity than the human AhR^68^. These results highlight the functional disparities that may exist in rodent model systems, resulting in a failure to accurately predict human AhR function in response to given ligands.

Finally, intestinal epithelial cells are simultaneously exposed to (i) gut microbiota-derived compounds, (ii) gut microbiota metabolites generated by the presence of the chemicals and (iii) the pollutant along with its potential degradation compound(s). This result indicates that pollutant exposure risks, based primarily on human biotransformation enzymes, may also take into account gut microbial processes, leading to more or less toxic compounds and/or microbial pro-inflammatory molecules. Depending on the pollutant and the intensity and frequency of exposure, gut microbiota could either protect host cells or enhance toxic and inflammatory responses.

## Conclusion

The present work highlights, for the first time, the impact of a panel of POPs and foodborne chemical families on human gut microbiota functions. We identified microbial volatiles and metabolic pathways that shifted after chemical exposure, leading to an imbalance in microbial activity. Finally, we showed that this gut microbiota-pollutant interplay might potentially lead to the establishment of a pro-inflammatory state in the gut. Therefore, chemical exposure risk assessment based primarily on human biotransformation enzymes might be underestimated.

## Materials and Methods

### Chemicals

Deltamethrin (pyrethroid insecticide), α-hexabromocyclododecane (α-HBCD), γ-hexabromocyclododecane (γ-HBCD) (BFRs), B[*a*]P (PAH), PAH Mix 3, methanol and dichloromethane were purchased from Sigma Aldrich (Saint-Quentin Fallavier, France). TCDD (PCDD) and PhIP (HCA) were purchased from LGC Standards (Molsheim, France). To avoid solubility issues in batches, all pollutants were dissolved in methanol (vehicle 1) except PAH Mix 3, which was purchased already dissolved in a methanol:dichloromethane (1:1) (v/v) solution (vehicle 2). The HBCD solution was an equal mixture of the α and γ isomers. The composition of the PAH Mix 3 solution is detailed in Supplementary Table 1. Chemicals and all contaminated effluents and materials were handled in an advised and safe manner with all necessary precautions.

### Experimental design

The pollutants were added at different concentrations (see below) into Hungate tubes along with a fecal microbiota suspension sampled from the continuous fermentor Mini Bioreactor Applikon® (Applikon, The Netherlands) following seven days of microbial stabilization. The fecal microbiota suspension contained *in vitro*-cultured feces collected from a human volunteer donor. Informed consent was obtained from the healthy volunteer. This was a non-interventional study with no additions to usual clinical care. According to French Health Public Law (CSP Art L 1121-1.1), such a protocol does not require approval of an ethics committee. The incubation volume (10 mL) was composed of one-fourth fecal microbiota suspension and three-fourths colon medium as previously described^16^.

The pollutant concentrations in batches were 0.005, 0.90, 2.60, 5, 21 and 38 µg/mL for TCDD, PhIP, HBCD, B[*a*]P, deltamethrin, and PAHs, respectively. Vehicle 1 and vehicle 2 were added at 0.5% (v/v) in batches. The concentration of methanol was kept below 1% (v/v) in medium to avoid potential microbial growth inhibition^69^. To take into account the impact of the vehicles on the fecal microbiota, batches with only vehicle 1 or 2 (vehicle 1 condition and vehicle 2 condition) at 0.5% (v/v) and batches with no pollutant and no vehicle (control condition) were added to the experimental design. Five replicates were assessed for all control and experimental conditions.

At the end (T24 hr) of the incubation step, samples dedicated to RNA extractions were immediately centrifuged at 2000 × *g* for 8 min. The pellets were then resuspended in 5 volumes of RNA*later*® (Fisher Scientific, Illkirch, France) and maintained at −80 °C until extraction. The remaining incubation medium was either directly maintained at −20 °C for SPME-GC-MS analysis or centrifuged at 2000 × g for 10 min (referred as FDS). The five FDS replicates were pooled and maintained at −20 °C until used to challenge TC7 cells.

### Microbial volatolome analysis

The volatolome analysis was performed as previously described by Defois *et al*.^16^. Briefly, the volatile compounds in the samples were analyzed *via* SPME-GC-MS. A volatile compound analysis was performed by GC-full scan MS (GC6890, MS5973N, Agilent). The volatiles were tentatively identified according to a comparison between their mass spectra and the NIST 14 mass spectral library and between published retention index (RI) values and the RI values of an internal databank. Peak areas of the volatile compounds were determined with a home-made automatic algorithm developed in Matlab R2014b (The MathWorks, Natick, USA). The data were processed using Statistica software (v.10) (StatSoft, Maisons-Alfort, France). T-tests (*p* < 0.05) were applied to datasets corresponding to each case-control study (exposed group vs vehicle group). In order to limit the risk of false positive results, the *p*-values of compounds selected by t-tests were then corrected for multiple testing: after the pre-selection of the n_i_ responding compounds (*p* < 0.05), a Bonferroni correction (*p* < 0.05/n_i_) was applied for each of the 6 case-control comparisons.

### RNA extraction, sequencing and analysis

RNA extraction and rRNA depletion were performed as previously described^16^. The metatranscriptome analysis was performed on the pooled rRNA-depleted RNA arising from five technical replicates.

Library construction (following TruSeq Stranded mRNA Sample Preparation, Illumina) and paired-end sequencing (MiSeq, 2 × 250 bp) were performed at Fasteris (Plan-les-Ouates, Switzerland). The paired-end sequences were assessed for quality with Trimmomatic^70^ and PRINSEQ^71^. and joined with fastq-join from the ea-utils software package^72^. The remaining rRNA sequences were removed from the data set using SortMeRNA (v. 2.0) software^73^. The UniRef50 gene family and GO slim term relative abundances in CPM were obtained using the HMP Unified Metabolic Analysis Network2 (HUMAnN2) software (v0.5)^74^. GO slim term-derived heatmaps were created using Shinyheatmap software^75^. Only genes with at least a 3-fold change between the pollutant and the vehicle conditions were analyzed.

### TC7 cell culture

TC7 cells (clone of the parenteral Caco-2 epithelial cell line) were kindly provided by Dr. Adeline Sivignon (M2iSH, Clermont-Ferrand, France). Caco-2 and TC7 cell lines are popularly used for studies on intestinal metabolism and absorption of various pharmaceutical and nutritional compounds^76,77^. TC7 cell line is a Caco-2 clone thus presenting numerous properties found in enterocytes such as cell polarization, brush border, cell junctions as well as cell metabolism enzymes. However, clone TC7 shows better homogeneity than the parental Caco line as well as more developed intercellular junctions. Also, the TC7 line has a faster metabolism than the Caco line leading to a complete differentiation of the cells under 14 days of culture compared to 21 days as required by the Caco line^61^. The cell culture medium consisted of Dulbecco’s Modified Eagle Medium with high glucose (4.5 g/L) supplemented with 10% fetal bovine serum, 1% glutamine, 1% non-essential amino acids (100X) and 1% penicillin-streptomycin (100X). DMEM and the added components were purchased from Gibco® (Fisher Scientific).

TC7 cells were maintained in 75-cm² flasks (Falcon™, Fisher Scientific) at 37 °C in a humidified atmosphere of 5% CO_2_. When the cells reached 80% confluence, the medium was removed from the dish, and cells were washed twice with 10 mL of PBS solution (Gibco, Fisher Scientific). The cells were then supplemented with 0.25% trypsin-EDTA (Gibco, Fisher Scientific) and left in the incubator for 5 min. Trypsin action was stopped by addition of 10 mL of complete medium, and the cell suspension was centrifuged for 5 min at 900 g. After removing the supernatant, the cell pellet was resuspended in complete medium, seeded directly into 12-well plates Nunclon™ (Fisher Scientific) (2.10^5^ cells/well) and maintained in culture for 14 days to allow complete cell differentiation before challenge^61^. The medium was changed three times a week beginning at the first week and every other day until day 14. TC7 cells were used between passages 30 and 41.

### Exposure of TC7 cells to fermentation-derived supernatants

Cells were treated for 4 hr with the FDS from each pollutant, vehicle and control conditions. Furthermore, TC7 cells were also exposed to fecal microbiota-free colon medium supplemented with each pollutant at its initial experimental concentration (microbiota-free condition). The supernatants were diluted 1:4 in DMEM because this dilution of the colon medium showed no cytotoxic effect on TC7 cells (results not shown). Finally, cellular control conditions (DMEM, dimethyl sulfoxide (DMSO) 10% (Eurobio, Les Ulis, France) and Interleukin (IL) 1 beta (IL-1β) (Sigma Aldrich) at 25 ng/mL) were included in the experimental design, and each condition was assessed in triplicate. DMSO and IL-1β were used as positive controls for the induction of toxicity (apoptosis and necrosis) and inflammation (cytokine release) in TC7 cells, respectively.

Following a 4-hr of exposure, the cellular supernatants were stored at −80 °C for cytokine release measurements, and the cell monolayers were harvested for apoptosis/necrosis detection. The results are the mean of three replicates, and statistical analyses were conducted using the Mann-Whitney *U*-test with GraphPad Prism 5 software (San Diego, CA, USA). The statistical significance was set at p < 0.05.

### Apoptosis detection

TC7 cell monolayers were washed twice with Dulbecco’s Phosphate-Buffered Saline (Gibco, Fisher Scientific) and treated with trypsin-EDTA 0.25%. Cells were recovered with cold Phosphate-Buffered Saline (Gibco, Fisher Scientific), centrifuged (500 g, 4 °C, 5 min) and subjected to the Annexin A5-FITC Kit (Beckman Coulter, Villepinte, France) following the manufacturer’s instructions. The assay combines Annexin A5 and Propidium Iodide staining, distinguishing viable cells from apoptotic cells and necrotic cells, respectively. Cells were then analyzed with a Cytomics FC 500 MPL flow cytometer.

### TC7 cytokine quantification

TC7 cell supernatants were centrifuged (1,000 g, 4 °C, 12 min), and the amount of released IL-8, Tumor Necrosis Factor alpha (TNFα) and IL-10 was determined using the Human IL-8 / CXCL8 ELISA Kit, Human TNF-Alpha ELISA Kit and Human IL-10 ELISA Kit (Sigma Aldrich), respectively. Cytokine concentrations were assessed according to the manufacturer’s instructions.

### Accession codes

All sequence data produced *via* RNA sequencing are available in the NCBI Sequence Read Archive, BioProject PRJNA416988, under accession no. SRP124200.

## Electronic supplementary material

Supplementary Information

Dataset 1

Dataset 2
