# Supplementary material for: Food Chemicals Disrupt Human Gut Microbiota Activity And Impact Intestinal Homeostasis As Revealed By In Vitro Systems
Source: Sci Rep. 2018 Jul 20;8:11006. doi: 10.1038/s41598-018-29376-9 (PMC6054606; doi:10.1038/s41598-018-29376-9)
Supplement: Supplementary file 1 — Supplementary Information [file 41598_2018_29376_MOESM1_ESM.pdf]

Supplementary Material

FOOD CHEMICALS DISRUPT HUMAN GUT MICROBIOTA ACTIVITY AND IMPACT INTESTINAL HOMEOSTASIS AS REVEALED BY *IN VITRO* SYSTEMS

Clémence Defois[1], Jérémy Ratel[2], Ghislain Garrait[1], Sylvain Denis[1], Olivier LeGoff[1], Jérémie Talvas[3,4], Pascale Mosoni[1], Erwan Engel[2], Pierre Peyret[1*]

[1]MEDIS, Université Clermont Auvergne, INRA, Clermont-Ferrand, France

[2]UR370 QuaPA, MASS Group, INRA, Saint-Genès-Champanelle, France

[3]UMR 1019, Unité de Nutrition Humaine, Equipe ECREIN, CLARA, Université Clermont Auvergne, Clermont-Ferrand, France

[4]UMR 1019, Unité de Nutrition Humaine, CRNH Auvergne, INRA, Clermont-Ferrand, France

* **Corresponding author**: Pierre Peyret
Tel: +33 (0)473178308; E-mail: pierre.peyret@uca.fr

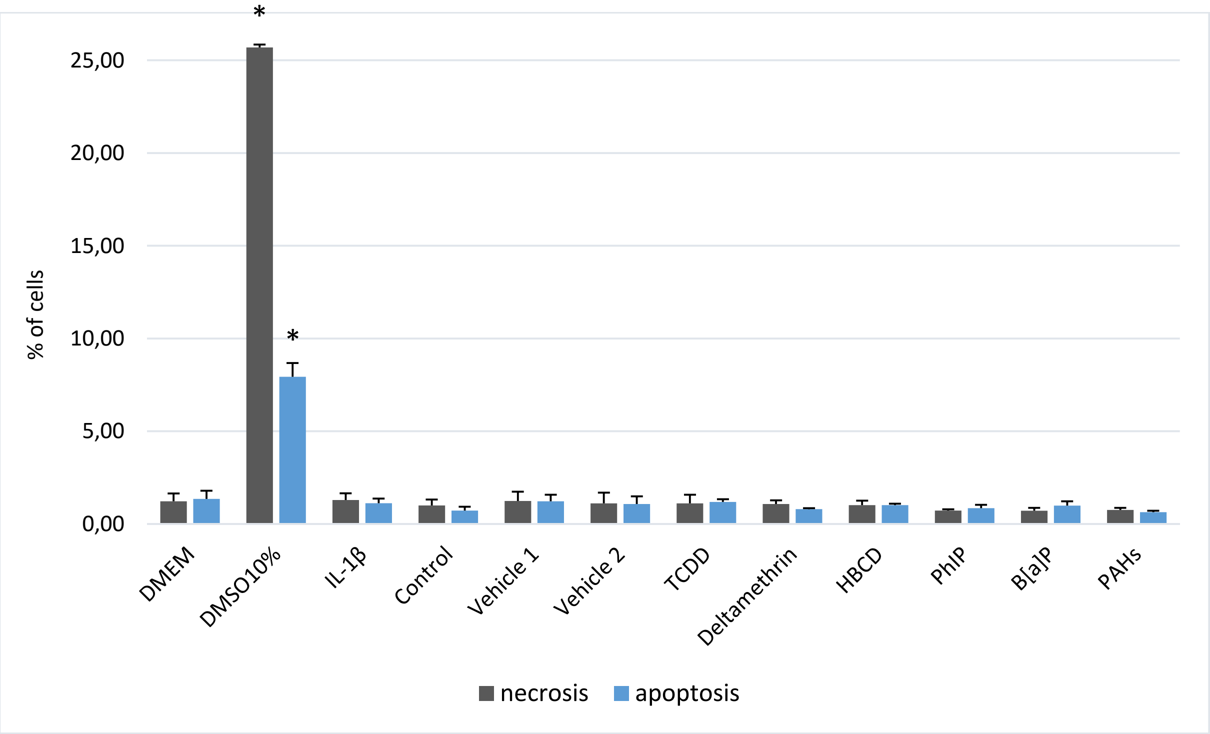

Supplementary Figure 1: Percentage of necrotic and apoptotic TC7 cells after 4 hr of FDS exposure. Values represent the mean of three replicates ± SEM. Significant variations were assessed using the Mann-Whitney test (p-value < 0.05). DMEM was a negative control for toxicity and inflammation, whereas 10% DMSO and IL-1β were positive controls for toxicity and inflammation in TC7 cells. Control: no pollutant and no vehicle; Vehicle 1: methanol; Vehicle 2: methanol:dichloromethane 1:1.

Supplementary Table 1. Composition of PAH mix 3 solution, proportion and PAH characteristics.

PAHs	proportion (%)	aromatic rings	molecular weight (g/mol)
Anthracene	1.3	3	178.23
Phenanthrene	1.3	3	178.23
Benz[*a*]anthracene	1.3	4	228.29
Chrysene	1.3	4	228.29
Fluoranthene	1.3	4	202.25
Pyrene	1.3	4	202.25
Benzo[*b*]fluoranthene	1.3	5	252.31
Benzo[*k*]fluoranthene	1.3	5	252.31
Benzo[*a*]pyrene	1.3	5	252.31
Indeno[1,2,3-*cd*]pyrene	1.3	6	276.34
Fluorene	2.6	3	166.22
Dibenz[*a,h*]anthracene	2.6	5	278.35
Benzo[*ghi*]perylene	2.6	6	276.33
1-Methylnaphthalene	13.2	2	142.20
2-Methylnaphthalene	13.2	2	142.20
Naphthalene	13.2	2	128.17
Acenaphthene	13.2	3	154.21
Acenaphthylene	26.3	3	152.20
